# 6-Azido-3-*O*-benzyl-6-de­oxy-*N*,*N*-diethyl-1,2-*O*-isopropyl­idene-d-*glycero*-α-d-*gluco*-heptofuran­uronamide

**DOI:** 10.1107/S1600536810048944

**Published:** 2010-12-04

**Authors:** S. F. Jenkinson, N. Oña, A. Romero, G. W. J. Fleet, A. L. Thompson, M. S. Pino-González

**Affiliations:** a Department of Organic Chemistry, Chemistry Research Laboratory, University of Oxford, Oxford OX1 3TA, England; b Dpto. Química Orgánica, Facultad de Ciencias, Universidad de Málaga, 29071 Málaga, Spain; c Department of Chemical Crystallography, Chemistry Research, Laboratory, University of Oxford, Oxford OX1 3TA, England

## Abstract

Reaction of 3-*O*-benzyl-1,2-*O*-isopropyl­idene-α-*xylo*-pentodialdo-1,4-furan­ose with *N*,*N*-diethyl-2-(dimethyl­sulfuranil­idene)acetamide gave stereoselectively an ep­oxy­amide, which was regioselectively opened by NaN_3_ in dimethyl formamide to give the title compound, C_21_H_30_N_4_O_6_. X-ray crystallography confirmed the relative stereochemistry of the title compound and the absolute configuration was determined by the use of d-glucose as the starting material. There are two mol­ecules in the asymmetric unit (*Z*′ = 2). The crystal structure consists of two types of chains of O—H⋯O hydrogen-bonded mol­ecules running parallel to the *b* axis, with each mol­ecule acting as a donor and acceptor of one hydrogen bond.

## Related literature

For the use of sulfur ylids to form ep­oxy­amides, see: Assiego *et al.* (2004 ▶); Jenkinson *et al.* (2009 ▶; López-Herrera *et al.* (1996 ▶, 1997 ▶); Oña *et al.* (2010 ▶); Pino-González *et al.* (2003 ▶, 2008 ▶); Valpuesta Fernández *et al.* (1990 ▶).
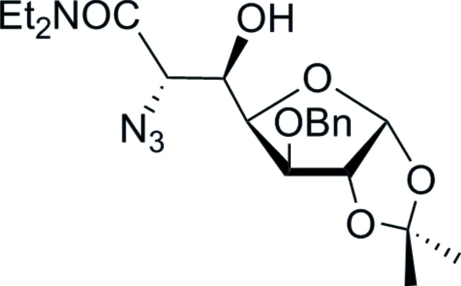


## Experimental

### 

#### Crystal data

C_21_H_30_N_4_O_6_
*M* *_r_* = 434.49
Monoclinic, 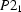

*a* = 13.0478 (4) Å
*b* = 10.5547 (3) Å
*c* = 16.5552 (6) Å
β = 97.0347 (11)°
*V* = 2262.75 (13) Å^3^
*Z* = 4
Mo *K*α radiation
μ = 0.09 mm^−1^
*T* = 150 K
0.20 × 0.20 × 0.20 mm

#### Data collection

Nonius KappaCCD diffractometer
Absorption correction: multi-scan (*DENZO*/*SCALEPACK*; Otwinowski & Minor, 1997 ▶) *T*_min_ = 0.92, *T*_max_ = 0.98
15625 measured reflections
5408 independent reflections
4270 reflections with *I* > 2σ(*I*)
*R*_int_ = 0.042

#### Refinement

*R*[*F* ^2^ > 2σ(*F* ^2^)] = 0.053
*wR*(*F* ^2^) = 0.122
*S* = 0.96
5408 reflections
559 parameters
1 restraint
H-atom parameters constrained
Δρ_max_ = 0.56 e Å^−3^
Δρ_min_ = −0.44 e Å^−3^

### 

Data collection: *COLLECT* (Nonius, 2001 ▶); cell refinement: *DENZO*/*SCALEPACK* (Otwinowski & Minor, 1997 ▶); data reduction: *DENZO*/*SCALEPACK*; program(s) used to solve structure: *SIR92* (Altomare *et al.*, 1994 ▶); program(s) used to refine structure: *CRYSTALS* (Betteridge *et al.*, 2003 ▶); molecular graphics: *CAMERON* (Watkin *et al.*, 1996 ▶); software used to prepare material for publication: *CRYSTALS*.

## Supplementary Material

Crystal structure: contains datablocks global, I. DOI: 10.1107/S1600536810048944/lh5169sup1.cif

Structure factors: contains datablocks I. DOI: 10.1107/S1600536810048944/lh5169Isup2.hkl

Additional supplementary materials:  crystallographic information; 3D view; checkCIF report

## Figures and Tables

**Table 1:** Hydrogen-bond geometry (Å, °)

*D*—H⋯*A*	*D*—H	H⋯*A*	*D*⋯*A*	*D*—H⋯*A*
O33—H331⋯O40^i^	0.84	2.39	3.046 (5)	135
O10—H101⋯O5^ii^	0.85	2.25	2.849 (5)	127

Symmetry codes: (i) 
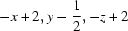
; (ii) 
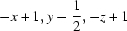
.

**Fig. 1.**
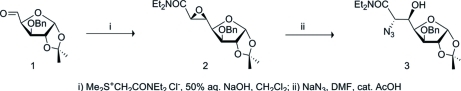
Synthetic Scheme

**Fig. 2.**
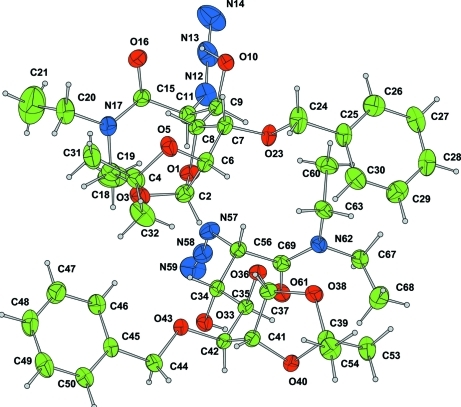
The title compound with displacement ellipsoids drawn at the 50% probability level. H atoms are shown as spheres of arbitrary radius.

**Fig. 3.**
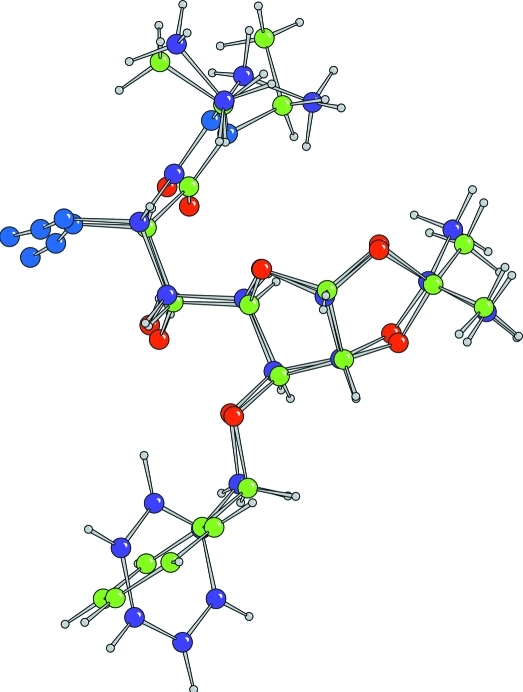
Overlay of the two molecules in the asymmetric unit.

**Fig. 4.**
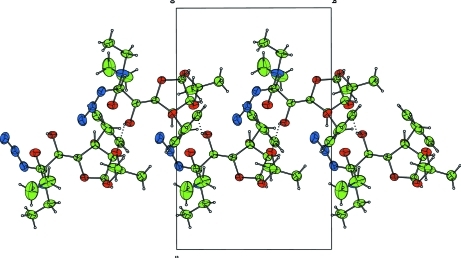
Packing diagram of the title compound projected along the a-axis. Hydrogen bonds are shown by dotted lines.

## supplementary crystallographic information

### Comment

The stereoselective formation of epoxyamides from monosacharides and sulfur ylids and the subsequent regioselective epoxide opening has been utilized in the formation of iminosugars such as the seven-membered ring azepanes (Oña *et al.*, 2010), pyrrolidines (Pino-González *et al.*, 2008), pipecolic acid derivatives (Pino-González *et al.*, 2008) and other monosaccharide derivatives as *C*-glycosides (López-Herrera *et al.*, 1997) and branched furanoses (Jenkinson *et al.*, 2009). In order to obtain new products, key intermediates in iminosugar synthesis, the reaction of semiprotected dialdose 1 with *N*,*N*-diethyl-2-(dimethylsulfuranylidene)acetamide was investigated.

Reaction of aldehyde 1, obtained from *D*-glucose, with the sulfur ylid gave epoxyamide 2 as the only product (Fig. 1). The subsequent regioselective opening of the epoxide with sodium azide, with acetic acid as a catalyst, gave the title compound 3. The product was confirmed, by both X-ray crystallography and the use of *D*-glucose as the starting material, to have the *D*-*glycero*-*D*-*gluco* stereochemistry (Fig. 2) arising from initial attack of the ylid on the *re* face of the aldehyde. This configuration can be predicted from a Felkin-Ahn model as corroborated for a number of aldehyde sugars with either free aldehydes or in cyclohemiacetalic form (Valpuesta Fernández *et al.*, 1990; López-Herrera *et al.*, 1996). In the present compound 1, the influence of 3-*O*-benzyl group plays a crucial role to obtain complete selectivity.

There are two crystallographically distinct molecules in the asymmetric unit which are related by a pseudo 2-fold rotation axis (Fig 2). When the two molecules are mapped they show good overlap for the majority of the structure (Fig. 3) with RMS deviations of 1.0604 on the positions, 0.0140 for the bonds and 35.7787 for the torsion angles. The major difference between the two molecules is the orientation of the aromatic ring. The crystal exists as chains of hydrogen bonded molecules lying parallel to the *b*-axis, with each molecule acting as a donor and an acceptor of one hydrogen bond (Fig. 4). Only classical hydrogen bonding is considered. For one of the molecules in the asymmetric unit the ethylamine moiety exhibits a greater degree of thermal motion than in the other.

### Experimental

The title compound was recrystallized from dichloromethane: m.p. 375–376 K; [α]_D_^29^ -2.28 (1.4, CH_2_Cl_2_).

### Refinement

In the absence of significant anomalous scattering, Friedel pairs were merged and the absolute configuration was assigned from the use of *D*-glucose as the starting material.

The H atoms were all located in a difference map, but those attached to carbon atoms were repositioned geometrically. The H atoms were initially refined with soft restraints on the bond lengths and angles to regularize their geometry (C—H in the range 0.93–0.98, O—H = 0.82 Å) and *U*_iso_(H) (in the range 1.2–1.5 times *U*_eq_ of the parent atom), after which the positions were refined with riding constraints.

### Crystal data

**Table:**

C_21_H_30_N_4_O_6_	*F*(000) = 928
*M**_r_* = 434.49	*D*_x_ = 1.275 Mg m^−^^3^
Monoclinic, *P*2_1_	Mo *K*α radiation, λ = 0.71073 Å
Hall symbol: P 2yb	Cell parameters from 4645 reflections
*a* = 13.0478 (4) Å	θ = 5–27°
*b* = 10.5547 (3) Å	µ = 0.09 mm^−^^1^
*c* = 16.5552 (6) Å	*T* = 150 K
β = 97.0347 (11)°	Block, colourless
*V* = 2262.75 (13) Å^3^	0.20 × 0.20 × 0.20 mm
*Z* = 4	

### Data collection

**Table:**

Nonius KappaCCD diffractometer	4270 reflections with *I* > 2σ(*I*)
graphite	*R*_int_ = 0.042
ω scans	θ_max_ = 27.5°, θ_min_ = 5.1°
Absorption correction: multi-scan (*DENZO*/*SCALEPACK*; Otwinowski & Minor, 1997)	*h* = −16→16
*T*_min_ = 0.92, *T*_max_ = 0.98	*k* = −13→12
15625 measured reflections	*l* = −21→21
5408 independent reflections	

### Refinement

**Table:**

Refinement on *F*^2^	Primary atom site location: structure-invariant direct methods
Least-squares matrix: full	Hydrogen site location: inferred from neighbouring sites
*R*[*F*^2^ > 2σ(*F*^2^)] = 0.053	H-atom parameters constrained
*wR*(*F*^2^) = 0.122	Method = Modified Sheldrick *w* = 1/[σ^2^(*F*^2^) + (0.04*P*)^2^ + 1.43*P*], where *P* = [max(*F*_o_^2^,0) + 2*F*_c_^2^]/3
*S* = 0.96	(Δ/σ)_max_ = 0.003
5408 reflections	Δρ_max_ = 0.56 e Å^−^^3^
559 parameters	Δρ_min_ = −0.44 e Å^−^^3^
1 restraint	

### Fractional atomic coordinates and isotropic or equivalent isotropic displacement parameters (Å^2^)

**Table:**

	*x*	*y*	*z*	*U*_iso_*/*U*_eq_	
O1	0.64815 (18)	0.4052 (2)	0.70138 (14)	0.0323	
C2	0.6244 (3)	0.5362 (3)	0.7050 (2)	0.0326	
O3	0.52450 (18)	0.5540 (3)	0.72847 (14)	0.0375	
C4	0.4683 (3)	0.6383 (4)	0.6712 (2)	0.0391	
O5	0.50934 (17)	0.6130 (3)	0.59658 (14)	0.0358	
C6	0.6160 (2)	0.5864 (4)	0.6164 (2)	0.0325	
C7	0.6459 (2)	0.4740 (3)	0.5665 (2)	0.0298	
C8	0.6189 (2)	0.3632 (3)	0.61879 (18)	0.0282	
C9	0.6733 (2)	0.2397 (3)	0.60611 (19)	0.0293	
O10	0.64964 (17)	0.1992 (2)	0.52424 (13)	0.0330	
C11	0.6496 (2)	0.1379 (3)	0.6682 (2)	0.0308	
N12	0.6996 (2)	0.0157 (3)	0.6550 (2)	0.0429	
N13	0.6700 (3)	−0.0417 (3)	0.5909 (2)	0.0477	
N14	0.6530 (4)	−0.1082 (4)	0.5375 (3)	0.0697	
C15	0.5332 (2)	0.1219 (3)	0.6642 (2)	0.0329	
O16	0.48494 (19)	0.0876 (3)	0.59857 (15)	0.0420	
N17	0.4860 (2)	0.1522 (4)	0.72949 (19)	0.0511	
C18	0.5406 (3)	0.1803 (5)	0.8104 (2)	0.0490	
C19	0.5848 (4)	0.0643 (5)	0.8548 (3)	0.0700	
C20	0.3693 (4)	0.1677 (7)	0.7180 (3)	0.0828	
C21	0.3191 (5)	0.0650 (7)	0.7556 (5)	0.1107	
O23	0.75398 (16)	0.4753 (2)	0.56203 (14)	0.0328	
C24	0.7781 (3)	0.5480 (4)	0.4939 (2)	0.0465	
C25	0.8930 (3)	0.5551 (4)	0.4966 (2)	0.0357	
C26	0.9436 (3)	0.4845 (4)	0.4428 (2)	0.0437	
C27	1.0507 (3)	0.4893 (4)	0.4466 (3)	0.0477	
C28	1.1072 (3)	0.5638 (4)	0.5032 (3)	0.0470	
C29	1.0577 (3)	0.6343 (4)	0.5561 (2)	0.0462	
C30	0.9513 (3)	0.6311 (4)	0.5530 (2)	0.0431	
C31	0.3567 (3)	0.6015 (5)	0.6614 (2)	0.0568	
C32	0.4884 (4)	0.7751 (4)	0.6964 (3)	0.0572	
O33	0.85567 (16)	0.4425 (2)	0.97984 (13)	0.0306	
C34	0.8314 (2)	0.4732 (3)	0.89580 (18)	0.0263	
C35	0.8804 (2)	0.5994 (3)	0.87875 (19)	0.0262	
O36	0.85475 (16)	0.6307 (2)	0.79389 (13)	0.0281	
C37	0.8626 (2)	0.7634 (3)	0.7846 (2)	0.0291	
O38	0.95612 (18)	0.7968 (3)	0.75537 (14)	0.0387	
C39	1.0141 (2)	0.8790 (3)	0.8134 (2)	0.0312	
O40	0.97597 (16)	0.8530 (2)	0.88876 (13)	0.0313	
C41	0.8693 (2)	0.8217 (3)	0.8702 (2)	0.0283	
C42	0.8428 (2)	0.7125 (3)	0.92412 (19)	0.0266	
O43	0.73311 (15)	0.7062 (2)	0.92145 (13)	0.0308	
C44	0.6988 (2)	0.7753 (4)	0.9865 (2)	0.0413	
C45	0.5827 (2)	0.7846 (3)	0.9760 (2)	0.0314	
C46	0.5195 (3)	0.7125 (4)	0.9202 (2)	0.0397	
C47	0.4129 (3)	0.7252 (4)	0.9147 (2)	0.0467	
C48	0.3685 (3)	0.8093 (5)	0.9639 (2)	0.0484	
C49	0.4308 (3)	0.8828 (4)	1.0175 (3)	0.0500	
C50	0.5372 (3)	0.8715 (4)	1.0240 (2)	0.0425	
C53	1.1262 (3)	0.8427 (4)	0.8215 (2)	0.0446	
C54	0.9960 (3)	1.0157 (3)	0.7890 (2)	0.0430	
C56	0.8605 (2)	0.3683 (3)	0.83931 (19)	0.0276	
N57	0.8102 (2)	0.2462 (3)	0.85459 (18)	0.0352	
N58	0.8394 (2)	0.1915 (3)	0.91974 (19)	0.0373	
N59	0.8557 (3)	0.1267 (3)	0.9747 (2)	0.0522	
C69	0.9774 (2)	0.3514 (3)	0.84793 (19)	0.0283	
O61	1.02124 (16)	0.3219 (3)	0.91594 (13)	0.0351	
N62	1.0296 (2)	0.3696 (3)	0.78426 (16)	0.0290	
C63	0.9861 (3)	0.4068 (3)	0.70130 (19)	0.0329	
C67	1.1427 (2)	0.3516 (4)	0.7969 (2)	0.0373	
C68	1.1979 (3)	0.4704 (5)	0.8269 (3)	0.0514	
C60	0.9706 (3)	0.2938 (4)	0.6450 (2)	0.0424	
H21	0.6795	0.5814	0.7411	0.0413*	
H61	0.6595	0.6608	0.6102	0.0413*	
H71	0.6058	0.4710	0.5120	0.0371*	
H81	0.5427	0.3517	0.6105	0.0355*	
H91	0.7502	0.2565	0.6153	0.0370*	
H111	0.6786	0.1682	0.7219	0.0400*	
H181	0.4892	0.2173	0.8431	0.0635*	
H182	0.5959	0.2426	0.8051	0.0631*	
H192	0.6168	0.0864	0.9086	0.1080*	
H191	0.5289	0.0039	0.8588	0.1082*	
H193	0.6370	0.0263	0.8254	0.1083*	
H202	0.3507	0.2449	0.7451	0.0996*	
H201	0.3400	0.1775	0.6607	0.0987*	
H211	0.2434	0.0744	0.7441	0.1711*	
H212	0.3432	0.0626	0.8148	0.1714*	
H213	0.3425	−0.0108	0.7288	0.1713*	
H242	0.7502	0.6334	0.4980	0.0610*	
H241	0.7465	0.5100	0.4427	0.0610*	
H261	0.9034	0.4324	0.4038	0.0557*	
H271	1.0850	0.4414	0.4092	0.0598*	
H281	1.1810	0.5652	0.5047	0.0609*	
H291	1.0965	0.6855	0.5947	0.0577*	
H301	0.9178	0.6804	0.5901	0.0536*	
H312	0.3196	0.6568	0.6209	0.0900*	
H311	0.3303	0.6102	0.7125	0.0902*	
H313	0.3496	0.5153	0.6438	0.0898*	
H322	0.4522	0.8297	0.6550	0.0915*	
H321	0.4641	0.7893	0.7485	0.0911*	
H323	0.5634	0.7908	0.7003	0.0914*	
H341	0.7558	0.4841	0.8852	0.0312*	
H351	0.9560	0.5943	0.8920	0.0324*	
H371	0.8030	0.7976	0.7487	0.0365*	
H411	0.8236	0.8951	0.8718	0.0377*	
H421	0.8789	0.7177	0.9801	0.0315*	
H442	0.7240	0.7335	1.0401	0.0541*	
H441	0.7271	0.8618	0.9869	0.0547*	
H461	0.5489	0.6538	0.8859	0.0497*	
H471	0.3707	0.6747	0.8759	0.0575*	
H481	0.2945	0.8160	0.9609	0.0622*	
H491	0.4014	0.9433	1.0501	0.0603*	
H501	0.5802	0.9223	1.0620	0.0551*	
H532	1.1655	0.9019	0.8600	0.0722*	
H531	1.1339	0.7567	0.8419	0.0727*	
H533	1.1517	0.8476	0.7682	0.0720*	
H541	1.0285	1.0712	0.8310	0.0684*	
H543	1.0243	1.0320	0.7386	0.0679*	
H542	0.9224	1.0313	0.7813	0.0679*	
H561	0.8352	0.3934	0.7826	0.0362*	
H632	1.0367	0.4644	0.6817	0.0422*	
H631	0.9207	0.4513	0.7039	0.0420*	
H671	1.1587	0.2815	0.8359	0.0484*	
H672	1.1632	0.3294	0.7432	0.0481*	
H683	1.2715	0.4572	0.8302	0.0791*	
H682	1.1808	0.4894	0.8820	0.0790*	
H681	1.1772	0.5395	0.7892	0.0790*	
H603	0.9415	0.3234	0.5905	0.0673*	
H602	1.0366	0.2524	0.6431	0.0672*	
H601	0.9232	0.2353	0.6685	0.0672*	
H331	0.9169	0.4160	0.9902	0.0479*	
H101	0.5900	0.1659	0.5196	0.0518*	

### Atomic displacement parameters (Å^2^)

**Table:**

	*U*^11^	*U*^22^	*U*^33^	*U*^12^	*U*^13^	*U*^23^
O1	0.0352 (12)	0.0328 (13)	0.0270 (12)	−0.0013 (10)	−0.0035 (9)	0.0019 (10)
C2	0.0323 (17)	0.0333 (18)	0.0311 (17)	−0.0026 (15)	−0.0002 (14)	0.0028 (14)
O3	0.0410 (13)	0.0453 (15)	0.0261 (12)	0.0036 (12)	0.0034 (10)	0.0031 (11)
C4	0.0401 (19)	0.050 (2)	0.0281 (17)	0.0059 (17)	0.0075 (14)	0.0039 (17)
O5	0.0290 (11)	0.0503 (16)	0.0287 (12)	0.0092 (11)	0.0060 (9)	0.0062 (11)
C6	0.0250 (15)	0.0374 (19)	0.0346 (18)	−0.0013 (14)	0.0018 (13)	0.0068 (15)
C7	0.0222 (14)	0.0347 (18)	0.0319 (17)	−0.0003 (14)	0.0013 (12)	0.0060 (15)
C8	0.0256 (15)	0.0340 (17)	0.0238 (15)	−0.0045 (14)	−0.0013 (12)	0.0009 (14)
C9	0.0235 (15)	0.0362 (19)	0.0277 (16)	−0.0034 (13)	0.0017 (12)	0.0028 (14)
O10	0.0314 (11)	0.0420 (14)	0.0263 (11)	−0.0067 (11)	0.0067 (9)	−0.0020 (11)
C11	0.0300 (16)	0.0332 (18)	0.0293 (17)	−0.0038 (14)	0.0037 (13)	0.0036 (14)
N12	0.0476 (18)	0.0355 (17)	0.0466 (19)	0.0057 (14)	0.0100 (15)	0.0080 (15)
N13	0.058 (2)	0.0339 (18)	0.055 (2)	0.0004 (17)	0.0242 (17)	0.0059 (18)
N14	0.108 (3)	0.040 (2)	0.067 (3)	0.001 (2)	0.034 (2)	−0.009 (2)
C15	0.0317 (16)	0.0377 (19)	0.0292 (17)	−0.0093 (15)	0.0031 (13)	0.0018 (15)
O16	0.0382 (13)	0.0553 (17)	0.0325 (13)	−0.0163 (12)	0.0043 (10)	−0.0033 (12)
N17	0.0348 (16)	0.087 (3)	0.0332 (17)	−0.0170 (17)	0.0091 (13)	−0.0074 (18)
C18	0.047 (2)	0.069 (3)	0.0325 (19)	−0.018 (2)	0.0115 (16)	−0.0099 (19)
C19	0.101 (4)	0.069 (3)	0.038 (2)	−0.033 (3)	−0.002 (2)	0.011 (2)
C20	0.057 (3)	0.132 (6)	0.066 (3)	−0.034 (3)	0.031 (2)	−0.018 (3)
C21	0.086 (4)	0.084 (5)	0.169 (7)	−0.012 (4)	0.045 (5)	−0.009 (5)
O23	0.0233 (10)	0.0404 (14)	0.0351 (12)	−0.0002 (10)	0.0053 (9)	0.0125 (11)
C24	0.0349 (18)	0.058 (3)	0.048 (2)	0.0060 (18)	0.0101 (16)	0.027 (2)
C25	0.0320 (17)	0.037 (2)	0.0392 (19)	0.0014 (15)	0.0098 (15)	0.0167 (16)
C26	0.051 (2)	0.037 (2)	0.044 (2)	−0.0025 (18)	0.0062 (17)	0.0039 (17)
C27	0.046 (2)	0.050 (2)	0.051 (2)	0.011 (2)	0.0215 (18)	0.009 (2)
C28	0.0343 (18)	0.053 (3)	0.056 (2)	0.0025 (18)	0.0127 (18)	0.019 (2)
C29	0.047 (2)	0.042 (2)	0.048 (2)	−0.0080 (18)	0.0006 (17)	0.0086 (19)
C30	0.050 (2)	0.040 (2)	0.041 (2)	0.0086 (18)	0.0115 (17)	0.0037 (17)
C31	0.0342 (18)	0.097 (4)	0.040 (2)	0.007 (2)	0.0108 (16)	0.011 (2)
C32	0.077 (3)	0.047 (3)	0.051 (3)	0.010 (2)	0.021 (2)	0.003 (2)
O33	0.0273 (11)	0.0394 (14)	0.0251 (11)	0.0026 (10)	0.0037 (9)	0.0038 (10)
C34	0.0197 (14)	0.0309 (17)	0.0282 (16)	0.0013 (13)	0.0024 (12)	0.0001 (14)
C35	0.0214 (13)	0.0323 (17)	0.0241 (15)	−0.0006 (13)	0.0001 (11)	−0.0002 (13)
O36	0.0311 (11)	0.0283 (12)	0.0241 (11)	−0.0009 (10)	0.0007 (8)	−0.0007 (9)
C37	0.0290 (15)	0.0296 (17)	0.0280 (16)	0.0002 (14)	0.0007 (12)	0.0006 (13)
O38	0.0428 (13)	0.0446 (15)	0.0304 (12)	−0.0145 (12)	0.0119 (10)	−0.0064 (11)
C39	0.0292 (16)	0.0349 (19)	0.0298 (17)	−0.0044 (14)	0.0052 (13)	−0.0019 (14)
O40	0.0277 (11)	0.0385 (13)	0.0280 (11)	−0.0082 (10)	0.0050 (9)	−0.0013 (10)
C41	0.0245 (14)	0.0286 (17)	0.0319 (17)	−0.0013 (13)	0.0034 (12)	−0.0028 (14)
C42	0.0196 (13)	0.0320 (17)	0.0275 (16)	0.0008 (13)	0.0005 (12)	−0.0038 (14)
O43	0.0218 (10)	0.0407 (14)	0.0307 (12)	−0.0016 (10)	0.0059 (8)	−0.0080 (11)
C44	0.0293 (16)	0.056 (2)	0.040 (2)	−0.0004 (17)	0.0096 (14)	−0.0167 (18)
C45	0.0286 (15)	0.0357 (19)	0.0319 (17)	−0.0009 (14)	0.0110 (13)	0.0028 (15)
C46	0.0322 (17)	0.047 (2)	0.0408 (19)	0.0026 (17)	0.0085 (14)	−0.0041 (17)
C47	0.0341 (18)	0.059 (3)	0.046 (2)	−0.0038 (18)	0.0029 (16)	0.002 (2)
C48	0.0291 (17)	0.066 (3)	0.051 (2)	0.0060 (19)	0.0112 (16)	0.016 (2)
C49	0.042 (2)	0.058 (3)	0.054 (2)	0.014 (2)	0.0201 (19)	0.000 (2)
C50	0.0389 (19)	0.049 (2)	0.042 (2)	0.0010 (18)	0.0143 (15)	−0.0085 (18)
C53	0.0336 (17)	0.056 (3)	0.046 (2)	0.0030 (18)	0.0125 (16)	0.0080 (19)
C54	0.050 (2)	0.034 (2)	0.046 (2)	0.0001 (17)	0.0091 (18)	0.0056 (17)
C56	0.0265 (15)	0.0303 (17)	0.0257 (16)	−0.0043 (13)	0.0016 (12)	0.0018 (14)
N57	0.0379 (15)	0.0300 (15)	0.0365 (16)	−0.0061 (13)	−0.0008 (12)	0.0003 (13)
N58	0.0395 (16)	0.0310 (16)	0.0418 (18)	−0.0037 (14)	0.0066 (13)	−0.0009 (15)
N59	0.074 (2)	0.0379 (19)	0.0445 (19)	−0.0024 (18)	0.0061 (17)	0.0123 (16)
C69	0.0293 (15)	0.0288 (17)	0.0267 (16)	0.0029 (14)	0.0025 (12)	−0.0012 (14)
O61	0.0308 (11)	0.0496 (15)	0.0239 (11)	0.0083 (11)	−0.0001 (9)	0.0041 (11)
N62	0.0288 (13)	0.0348 (15)	0.0237 (13)	0.0020 (12)	0.0045 (10)	0.0013 (12)
C63	0.0378 (17)	0.0365 (19)	0.0250 (16)	0.0042 (15)	0.0059 (13)	0.0030 (14)
C67	0.0282 (16)	0.053 (2)	0.0312 (17)	−0.0003 (16)	0.0045 (13)	−0.0016 (17)
C68	0.0390 (19)	0.063 (3)	0.053 (2)	−0.010 (2)	0.0071 (17)	0.001 (2)
C60	0.0452 (19)	0.050 (2)	0.0302 (18)	0.0101 (18)	−0.0017 (15)	−0.0052 (17)

### Geometric parameters (Å, °)

**Table:**

O1—C2	1.420 (4)	O33—C34	1.426 (4)
O1—C8	1.444 (4)	O33—H331	0.844
C2—O3	1.418 (4)	C34—C35	1.518 (5)
C2—C6	1.551 (5)	C34—C56	1.527 (5)
C2—H21	0.999	C34—H341	0.987
O3—C4	1.435 (4)	C35—O36	1.442 (4)
C4—O5	1.430 (4)	C35—C42	1.523 (5)
C4—C31	1.496 (5)	C35—H351	0.986
C4—C32	1.517 (6)	O36—C37	1.415 (4)
O5—C6	1.419 (4)	C37—O38	1.411 (4)
C6—C7	1.523 (5)	C37—C41	1.538 (5)
C6—H61	0.981	C37—H371	0.987
C7—C8	1.521 (5)	O38—C39	1.437 (4)
C7—O23	1.422 (4)	C39—O40	1.426 (4)
C7—H71	0.987	C39—C53	1.503 (5)
C8—C9	1.511 (5)	C39—C54	1.509 (5)
C8—H81	0.994	O40—C41	1.427 (4)
C9—O10	1.419 (4)	C41—C42	1.524 (5)
C9—C11	1.545 (4)	C41—H411	0.980
C9—H91	1.011	C42—O43	1.429 (3)
O10—H101	0.848	C42—H421	0.989
C11—N12	1.474 (5)	O43—C44	1.417 (4)
C11—C15	1.522 (4)	C44—C45	1.506 (4)
C11—H111	0.977	C44—H442	1.010
N12—N13	1.241 (5)	C44—H441	0.985
N13—N14	1.129 (5)	C45—C46	1.388 (5)
C15—O16	1.241 (4)	C45—C50	1.393 (5)
C15—N17	1.346 (5)	C46—C47	1.390 (5)
N17—C18	1.469 (5)	C46—H461	0.952
N17—C20	1.520 (6)	C47—C48	1.379 (6)
C18—C19	1.506 (7)	C47—H471	0.955
C18—H181	0.992	C48—C49	1.369 (6)
C18—H182	0.988	C48—H481	0.963
C19—H192	0.965	C49—C50	1.384 (5)
C19—H191	0.978	C49—H491	0.946
C19—H193	0.970	C50—H501	0.955
C20—C21	1.446 (9)	C53—H532	0.990
C20—H202	0.975	C53—H531	0.970
C20—H201	0.984	C53—H533	0.981
C21—H211	0.988	C54—H541	0.967
C21—H212	0.993	C54—H543	0.969
C21—H213	0.982	C54—H542	0.966
O23—C24	1.431 (4)	C56—N57	1.482 (4)
C24—C25	1.497 (5)	C56—C69	1.524 (4)
C24—H242	0.978	C56—H561	0.992
C24—H241	0.982	N57—N58	1.241 (4)
C25—C26	1.388 (5)	N58—N59	1.137 (4)
C25—C30	1.387 (5)	C69—O61	1.239 (4)
C26—C27	1.392 (6)	C69—N62	1.338 (4)
C26—H261	0.955	N62—C63	1.474 (4)
C27—C28	1.368 (6)	N62—C67	1.476 (4)
C27—H271	0.952	C63—C60	1.512 (5)
C28—C29	1.370 (6)	C63—H632	0.981
C28—H281	0.960	C63—H631	0.980
C29—C30	1.383 (5)	C67—C68	1.501 (6)
C29—H291	0.937	C67—H671	0.988
C30—H301	0.951	C67—H672	0.987
C31—H312	0.972	C68—H683	0.965
C31—H311	0.957	C68—H682	0.986
C31—H313	0.956	C68—H681	0.976
C32—H322	0.972	C60—H603	0.986
C32—H321	0.968	C60—H602	0.971
C32—H323	0.986	C60—H601	0.988
			
C2—O1—C8	107.8 (2)	C34—O33—H331	111.4
O1—C2—O3	110.7 (3)	O33—C34—C35	109.5 (2)
O1—C2—C6	106.5 (3)	O33—C34—C56	113.0 (3)
O3—C2—C6	104.7 (2)	C35—C34—C56	112.4 (2)
O1—C2—H21	110.3	O33—C34—H341	107.2
O3—C2—H21	113.0	C35—C34—H341	107.4
C6—C2—H21	111.4	C56—C34—H341	107.0
C2—O3—C4	108.3 (2)	C34—C35—O36	109.2 (2)
O3—C4—O5	103.9 (3)	C34—C35—C42	115.3 (2)
O3—C4—C31	108.8 (3)	O36—C35—C42	104.5 (3)
O5—C4—C31	108.7 (3)	C34—C35—H351	110.1
O3—C4—C32	110.6 (3)	O36—C35—H351	109.6
O5—C4—C32	110.2 (3)	C42—C35—H351	107.9
C31—C4—C32	114.2 (4)	C35—O36—C37	108.8 (2)
C4—O5—C6	107.3 (2)	O36—C37—O38	111.2 (3)
C2—C6—O5	103.9 (3)	O36—C37—C41	107.0 (3)
C2—C6—C7	104.6 (3)	O38—C37—C41	105.2 (2)
O5—C6—C7	109.7 (3)	O36—C37—H371	111.4
C2—C6—H61	113.5	O38—C37—H371	110.7
O5—C6—H61	112.4	C41—C37—H371	111.1
C7—C6—H61	112.2	C37—O38—C39	109.2 (2)
C6—C7—C8	101.5 (3)	O38—C39—O40	104.9 (2)
C6—C7—O23	110.0 (3)	O38—C39—C53	109.5 (3)
C8—C7—O23	109.7 (3)	O40—C39—C53	108.2 (3)
C6—C7—H71	112.3	O38—C39—C54	110.2 (3)
C8—C7—H71	111.2	O40—C39—C54	110.9 (3)
O23—C7—H71	111.7	C53—C39—C54	112.7 (3)
C7—C8—O1	104.5 (3)	C39—O40—C41	106.9 (2)
C7—C8—C9	116.0 (3)	C37—C41—O40	103.6 (2)
O1—C8—C9	108.8 (2)	C37—C41—C42	104.2 (3)
C7—C8—H81	108.2	O40—C41—C42	109.5 (2)
O1—C8—H81	108.2	C37—C41—H411	112.1
C9—C8—H81	110.8	O40—C41—H411	113.1
C8—C9—O10	110.0 (3)	C42—C41—H411	113.5
C8—C9—C11	111.8 (3)	C41—C42—C35	101.2 (2)
O10—C9—C11	112.8 (3)	C41—C42—O43	108.3 (2)
C8—C9—H91	107.6	C35—C42—O43	109.3 (2)
O10—C9—H91	107.0	C41—C42—H421	113.1
C11—C9—H91	107.3	C35—C42—H421	111.2
C9—O10—H101	107.6	O43—C42—H421	113.1
C9—C11—N12	112.4 (3)	C42—O43—C44	111.2 (2)
C9—C11—C15	109.2 (3)	O43—C44—C45	110.7 (3)
N12—C11—C15	110.8 (3)	O43—C44—H442	110.1
C9—C11—H111	106.9	C45—C44—H442	110.2
N12—C11—H111	107.0	O43—C44—H441	109.2
C15—C11—H111	110.5	C45—C44—H441	108.2
C11—N12—N13	117.1 (3)	H442—C44—H441	108.5
N12—N13—N14	169.4 (4)	C44—C45—C46	123.2 (3)
C11—C15—O16	117.8 (3)	C44—C45—C50	118.0 (3)
C11—C15—N17	119.4 (3)	C46—C45—C50	118.8 (3)
O16—C15—N17	122.7 (3)	C45—C46—C47	119.9 (4)
C15—N17—C18	124.1 (3)	C45—C46—H461	120.3
C15—N17—C20	118.4 (3)	C47—C46—H461	119.8
C18—N17—C20	117.3 (3)	C46—C47—C48	120.9 (4)
N17—C18—C19	113.2 (4)	C46—C47—H471	118.7
N17—C18—H181	106.9	C48—C47—H471	120.5
C19—C18—H181	107.4	C47—C48—C49	119.3 (3)
N17—C18—H182	109.3	C47—C48—H481	120.5
C19—C18—H182	110.2	C49—C48—H481	120.3
H181—C18—H182	109.8	C48—C49—C50	120.8 (4)
C18—C19—H192	110.5	C48—C49—H491	120.2
C18—C19—H191	108.6	C50—C49—H491	119.1
H192—C19—H191	109.7	C45—C50—C49	120.4 (4)
C18—C19—H193	110.0	C45—C50—H501	119.2
H192—C19—H193	108.1	C49—C50—H501	120.4
H191—C19—H193	109.9	C39—C53—H532	108.6
N17—C20—C21	111.4 (6)	C39—C53—H531	109.1
N17—C20—H202	109.5	H532—C53—H531	110.1
C21—C20—H202	106.1	C39—C53—H533	109.8
N17—C20—H201	113.3	H532—C53—H533	109.9
C21—C20—H201	110.6	H531—C53—H533	109.3
H202—C20—H201	105.5	C39—C54—H541	110.3
C20—C21—H211	109.7	C39—C54—H543	109.6
C20—C21—H212	109.8	H541—C54—H543	109.4
H211—C21—H212	112.4	C39—C54—H542	108.6
C20—C21—H213	103.5	H541—C54—H542	109.4
H211—C21—H213	110.7	H543—C54—H542	109.4
H212—C21—H213	110.3	C34—C56—N57	112.2 (3)
C7—O23—C24	111.2 (2)	C34—C56—C69	110.4 (3)
O23—C24—C25	108.6 (3)	N57—C56—C69	110.0 (3)
O23—C24—H242	108.7	C34—C56—H561	107.8
C25—C24—H242	109.4	N57—C56—H561	106.9
O23—C24—H241	110.6	C69—C56—H561	109.5
C25—C24—H241	111.1	C56—N57—N58	117.2 (3)
H242—C24—H241	108.4	N57—N58—N59	169.2 (4)
C24—C25—C26	120.5 (4)	C56—C69—O61	117.4 (3)
C24—C25—C30	120.9 (4)	C56—C69—N62	120.7 (3)
C26—C25—C30	118.6 (3)	O61—C69—N62	121.9 (3)
C25—C26—C27	120.3 (4)	C69—N62—C63	126.7 (3)
C25—C26—H261	118.6	C69—N62—C67	117.9 (3)
C27—C26—H261	121.1	C63—N62—C67	115.5 (3)
C26—C27—C28	120.4 (4)	N62—C63—C60	111.8 (3)
C26—C27—H271	120.0	N62—C63—H632	106.0
C28—C27—H271	119.6	C60—C63—H632	109.2
C27—C28—C29	119.6 (4)	N62—C63—H631	109.0
C27—C28—H281	118.8	C60—C63—H631	110.7
C29—C28—H281	121.6	H632—C63—H631	110.0
C28—C29—C30	120.9 (4)	N62—C67—C68	111.7 (3)
C28—C29—H291	119.4	N62—C67—H671	108.5
C30—C29—H291	119.7	C68—C67—H671	110.7
C25—C30—C29	120.3 (4)	N62—C67—H672	106.3
C25—C30—H301	119.6	C68—C67—H672	108.9
C29—C30—H301	120.1	H671—C67—H672	110.6
C4—C31—H312	108.3	C67—C68—H683	109.4
C4—C31—H311	109.3	C67—C68—H682	108.8
H312—C31—H311	110.4	H683—C68—H682	108.2
C4—C31—H313	109.7	C67—C68—H681	109.2
H312—C31—H313	109.9	H683—C68—H681	109.9
H311—C31—H313	109.2	H682—C68—H681	111.3
C4—C32—H322	108.6	C63—C60—H603	108.8
C4—C32—H321	109.2	C63—C60—H602	108.8
H322—C32—H321	110.6	H603—C60—H602	111.0
C4—C32—H323	108.4	C63—C60—H601	107.1
H322—C32—H323	109.8	H603—C60—H601	111.7
H321—C32—H323	110.2	H602—C60—H601	109.3

### Hydrogen-bond geometry (Å, °)

**Table:**

*D*—H···*A*	*D*—H	H···*A*	*D*···*A*	*D*—H···*A*
C2—H21···O36	1.00	2.40	3.333 (5)	155
C7—H71···O16^i^	0.99	2.39	3.268 (5)	147
C18—H182···O1	0.99	2.58	3.388 (5)	139
C31—H312···O10^i^	0.97	2.52	3.233 (5)	130
C42—H421···O61^ii^	0.99	2.31	3.216 (5)	152
O33—H331···O40^iii^	0.84	2.39	3.046 (5)	135
O33—H331···O61	0.84	2.18	2.822 (5)	133
O10—H101···O5^iv^	0.85	2.25	2.849 (5)	127
O10—H101···O16	0.85	2.17	2.858 (5)	138

Symmetry codes: (i) −*x*+1, *y*+1/2, −*z*+1; (ii) −*x*+2, *y*+1/2, −*z*+2; (iii) −*x*+2, *y*−1/2, −*z*+2; (iv) −*x*+1, *y*−1/2, −*z*+1.
